# Benefits of Fermented Papaya in Human Health

**DOI:** 10.3390/foods11040563

**Published:** 2022-02-16

**Authors:** Mariana Leitão, Tatiana Ribeiro, Pablo A. García, Luisa Barreiros, Patrícia Correia

**Affiliations:** 1 Pharmacy Faculty, University of Salamanca, 37007 Salamanca, Spain; 2 Research Centre on Health and Environment, Department of Pharmacy, School of Allied Health Sciences, Polytechnic Institute of Porto, 4200-072 Porto, Portugal; tatiribeiro96@gmail.com (T.R.); pcc@ess.ipp.pt (P.C.); 3 Department of Pharmaceutical Sciences, Pharmaceutical Chemistry Section, Faculty of Pharmacy, IBSAL, University of Salamanca, 37007 Salamanca, Spain; pabloagg@usal.es; 4 School of Allied Health Sciences, Polytechnic Institute of Porto, 4200-072 Porto, Portugal; lsb@ess.ipp.pt; 5 LAQV, REQUIMTE, Department of Chemical Sciences, Faculty of Pharmacy, University of Porto, 4050-313 Porto, Portugal

**Keywords:** fermented food, fermented papaya, health benefits, oxidative stress

## Abstract

Fermented foods have been used for several years all over the world, due to their unique nutritional characteristics and because fermentation promotes conservation and food security. Moreover, fermented foods and beverages have a strong impact on human gut microbiota. Papaya is the fruit of the *Carica papaya* plant, traditionally used as a medicinal fruit, but there are also references to the use of the fermented form of this fruit. The main purpose of this review is to provide an improved understanding of fermented papaya nutritional and health applications. A literature search was conducted in the PubMed and Google Scholar databases. Both in vitro and in vivo studies were included. According to the retrieved studies, fermented papaya has proven to be an excellent antioxidant and an excellent nutraceutical adjuvant in combined therapies against several diseases, such as Alzheimer’s disease, allergic reactions, anticancer activity, and anemias. Therefore, it is concluded that fermented papaya has many benefits for human health and can be used as prevention or aid in the treatment of various diseases.

## 1. Introduction

Fermentation is a central metabolism process in which an organism, mainly yeasts and some bacteria, converts sugars and starches into alcohol (alcoholic fermentation) or carboxylic acid (lactic or acetic fermentation), oxygen-free or -limited conditions. During this conversion, an intermediate product is formed, for instance, pyruvate (or acetaldehyde), produced from glucose metabolism [1,2]. The most famous application of this process is in the field of food and nutrition.

Fermented foods are defined as foods and beverages produced by enzymatic reactions with controlled microbial growth, promoting conservation and food security [3]. Fermentation is a food-preserving method in many parts of the world, particularly in Asia and in Africa, because it inhibits the growth of pathogenic bacteria even without refrigeration or other preserving methods [3]. This process is also used in other contexts. For instance, in regions where the safety of water supplies cannot be assured, fermentation contributes to reduce the risk of waterborne diseases [4,5]. Fermentation also can improve food’s organoleptic characteristics, such as taste and texture [3]. For example, olives are fermented to remove the bitter phenolic compounds [6]. Fermentation usually improves final foods’ nutritional value in contrast to processing methods, such as pasteurization, commonly used in today’s food industry. Fermented foods have a higher fiber content, essential fatty acids, amino acids, vitamins, and minerals [7].

Fermented products have been used for several years all over the world due to their nutritional characteristics, and there are several scientific evidences about the advantages of fermented food. Fermented milk products, such as yogurt, have been recognized as healthy foods since ancient times [1], and although this kind of food has been consumed since the beginning of civilization, its popularity has increased in recent times, essentially due to its therapeutic potential. More than a century ago, the Nobel prize winner Elie Metchnikoff (1845–1916) affirmed that fermented food, such as yogurt, can increase health and delay senility [8]. Metchnikoff’s concepts are currently supported by considerable evidence showing that lactic fermentation inhibits growth, survival, and production of toxins by some pathogenic and toxicogenic bacteria [9]. Another example is the consumption of an ancient Japanese fermented soy dish called natto that seems to increase vitamin K2 (menaquinone) concentrations in consumers, with several improvements in the health of the bones and heart [10]. Scientific evidence also suggests that the inclusion of fermented food in the diet can reduce the risk of cardiovascular disease [11] and the development of type 2 diabetes [7]. The National Institute of Nutrition’s 2010 suggests in the “Dietary Guidelines for Indians” that pregnant women should eat more fermented foods [1].

*Carica papaya*, also known as papaya or pawpaw, belongs to the *Caricaceae* family that is divided into four genera spread around the world. The genus *Carica* Linn. is the most cultivated and best-known species [12,13]. The papaya taxonomical classification includes kingdom (*Plantae*); order (*Brassicales*); family (*Caricaceae*); genus (*Carica*); and species (*Carica papaya*) [13]. Papaya was also named as “the fruit of the angels” by Christopher Columbus or as “a fruit of long life” [12]. Papaya is believed to be native to southern Mexico and neighboring Central America. It is currently widely planted in Brazil, Hawaii, Florida, South Africa, India, Australia, and others. Brazil is the leading world producer, with papaya as its most economically relevant fruit within the *Caricaceae* family [14].

Papaya’s main carbohydrates are simple sugars, as in ripe papaya, which contains 48% (*w*/*w*) sucrose, 30% (*w*/*w*) glucose, and 22% (*w*/*w*) fructose [14]; but the fruit is also rich in food fibers, which increases intestinal motility, which is enhanced with papain enzyme when it is present in large quantities [15]. Papaya contains a broad spectrum of phytochemicals, including enzymes (in latex), alkaloids (in leaves), phenolic compounds (in pulp and leaves), and carotenoid compounds and glucosinolates (in pulp and seeds). This fruit also contains large amounts of the least active pro-vitamin A, β-cryptoxanthin [16], and other micronutrients, such as sodium, calcium, phosphorus, zinc, iron, copper, and manganese, in a considerable amount [17]. For instance, in addition to phenolics (for example, vitamin K) and carotenoids (specifically pro-vitamin A), papaya’s pulp is also rich in magnesium, potassium, ascorbic acid (vitamin C), vitamin E, and B complex vitamins (such as pantothenic acid and folate). Lycopene, the main pigment in red pulp papaya, has important health implications as a strong antioxidant due to its great capacity for scavenging free radicals among carotenoids, closely followed by β-cryptoxanthin and β-carotene [18]. Seeds are rich in phenolic compounds, including benzyl isothiocyanate, glucosinolates, β-carotene, and carotenoids [19,20]. In terms of organic acids present in papaya, there is almost the same amount of malic and citric acids and smaller amounts of ascorbic acid and α-ketoglutaric acid [21]. It is recognized that there are some factors affecting papaya’s content: carotenoid and ascorbic acid content increase with ripeness; longer day lengths and higher light intensities in summer can increase fruit’s concentrations of ascorbic acid and its precursor, glucose [18]. Different parts of papaya have been used in folk medicine to treat various diseases, especially diabetes, cancer, and cardiovascular and infectious diseases. Some studies also show that papaya can also be used to prevent sickle cell anemia [22]. Carotenoids, phenolic compounds, and glucosinolates have attracted considerable interest in anticancer studies. These compounds may act via multiple mechanisms, such as cancer cell signaling, proliferation, apoptosis, migration, invasion, as well as angiogenesis and carcinogen elimination [23,24]. Usually, only papaya pulp is consumed, and ripe fruit is a carminative, diuretic, expectorant, sedative, and has preventive action against dysentery, skin diseases, psoriasis, and ringworm. The unripe fruit is used as a remedy for ulcers and impotence, reducing menstrual irregularities, and promoting natural menstruation flow in women [25]. The low-calory content (32 kcal/100 g of ripe fruit) and high nutritive value make papaya a preferred and excellent dietary product for weight-reducing regimes and diabetic patients [17]. Papaya juice helps in relieving colon infections and gastrointestinal maladies, such as dyspeptic and celiac disease, whose patients cannot digest wheat protein gliadin but can tolerate it if treated with crude papain [25]. In fact, two important compounds of papaya are chymopapain and papain, which are widely useful for digestive disorders and disturbance of the gastrointestinal tract. Physicochemical characterization and fatty acid composition analysis of crude oil extracted from papaya seeds showed high content of unsaturated fatty acids (78.17%), most of them monounsaturated fatty acids (71.89%). Therefore, papaya seed oil has a beneficial monounsaturated fatty acid profile and may have potential use in nutrition. Additionally, papaya seeds can also have another nutritional added value because they are a rich source of proteins (27.3–28.3%), lipids (28.2–30.7%), and crude fibers (19.1–22.6%) [26]. Furthermore, lipids with high monounsaturated fatty acid content are used in emollient skincare products, such as bath oils, hair conditioners, and makeup, which enhances seed oil potential in pharmaceuticals, specifically in dermo-cosmetics [26]. Both seed coat and papaya oil possess reasonable antioxidant properties, which emphasizes their nutritional potential and health benefits. The oil could also be useful for biofuel purposes, and seed coat may be used in the development of edible coating or packaging materials [27]. Papaya latex is very useful for healing dyspepsia, diarrhea, bleeding hemorrhoids, and whooping cough, and it is also externally applied to treat skin burns [17]. The green leaves of the tree are a source of essential nutrients, while the yellow ones provide mostly iron, which means that they can be used against anemia, tuberculosis, and growth disorders. It is also described that leaves with high iron content may have a synergistic action to reduce enlarged spleen and liver, and they are used to remove snakebite poison [17]. Malaria is also treated by using papaya leaves in some countries, such as Indonesia [28], probably related to the presence of antiplasmodial alkaloids in the leaves [29]. Leaves have also an important role in blood coagulation, the functioning of the heart and nervous system, and normal muscles movement [30]. Papaya leaves have also shown anti-hyperglycemic and hypolipidemic effects. A study carried out to evaluate the hypoglycemic effect of the aqueous extract of papaya leaves in streptozotocin-induced diabetic rats showed that there was a significant decrease in body weight in diabetic rats. After the administration of different doses of the aqueous extract of papaya to diabetic rats for 30 days, a significant decrease in blood glucose levels was observed. Moreover, significant decreases in serum cholesterol and triacylglycerol levels were observed in comparison to untreated diabetic rats after a 4-week administration of 3 g/100 mL of papaya leaf extract to diabetic rats [31]. The whole plant has high medicinal value as a result of its broad spectrum of vitamins, enzymes, and other active compounds [13].

Due to the already reported and numerous benefits of papaya and to the growing offer of different supplement brands with fermented papaya, such as the Fermented Papaya Product^®^ (FPP^®^), it is relevant to recognize the important of fermented papaya in human health. FPP^®^ is owned by Osato Research Institute in Japan and sold under the commercial name Immun’Âge^®^. This product is made with non-genetically modified *Carica papaya* and follows food safety standards (ISO 9001: 2000, ISO 14001: 2004, and ISO 22000: 2005). The final product is sold in granulated form. FPP^®^ is commercialized as a functional food in several countries (Japan, United States, and some European countries). FPP^®^ is made with wild, unripe *Carica papaya* Linn. fermented with *Enterococcus faecalis*, followed by fermentation with *Aspergillus oryzae*. These processes are performed at room temperature under aerobic conditions [8]. The therapeutic activity of FPP^®^ may be due to the formation of new bioactive compounds resulting from the fermentation process. With fermentation, polyphenols are converted into compounds of lower molecular weight and, consequently, with enhanced therapeutic activity [32,33]. In fact, the chemical analysis by spectroscopy and chromatography methods revealed the presence of low molecular weight phenolic acids (quinic acid, shikimic acid, and 2,5 dihydroxybenzoic acid) in fermented papaya [34].

Thus, the primary aim of this review is to provide an improved understanding of fermented papaya nutritional and health applications described in the literature.

## 2. Materials and Methods

Data were gathered from February to March 2021, using PubMed and Google Scholar databases, with the search terms “fermented papaya”, “Fermented Papaya Preparation”, “FPP^®^”, “Fermented papaya extracts”, “*Carica papaya*”, “Papaya”, “fermented food”, “fermented fruit”, and “fermented preparation”. The search was also carried out with the same search terms in Portuguese, but no further results were found. The articles admitted for analysis were published during the last twenty years, and included clinical studies (in vivo, in vitro, observational studies), review articles, and case reports, all of them in the English language. All abstracts were read in order to exclude the articles that were out of the scope of this study’s subject. The remaining articles were fully read, and then, data were collected, dividing the main subject into four categories: immunomodulatory, antioxidant, and anticancer properties; congenital/acquired hemolytic anemias; antidiabetic and antidislipidemic properties; and skin benefits and wound-healing properties.

## 3. Results and Discussion

Most studies on fermented papaya focus on its immunomodulatory, antioxidant, anticancer, and anti-inflammatory properties. However, there are also many studies that mention its antidiabetic and antidislipidemic properties and even its usefulness in anemias. Fermented papaya can also be applied in the dermo-cosmetic area due to its skin benefits.

### 3.1. Immunomodulatory, Antioxidant and Anticancer Properties

The main immunomodulatory, antioxidant, and anticancer effects of the fermented papaya are summarized in Table 1.

A study performed with FPP^®^ showed that this product has immunomodulatory and antioxidant properties in macrophages cell line RAW 264.7. When cells are treated only with FPP^®^ extracts, no significant differences in nitric oxide production are observed. However, when cells had been previously treated with interferon-γ (IFN-γ), both low molecular weight and high molecular weight FPP^®^ extracts increased the enzyme iNOS (nitric oxide synthase) activity, also increasing levels of nitrite and nitrate. Besides, when IFN-γ is present, the low and high molecular weight FPP^®^ extracts stimulate the secretion of tumor necrosis factor-α (TNF-α). It was also observed that the low molecular weight FPP^®^ extract has a greater capacity to scavenging superoxide anions when compared to high molecular weight FPP^®^ extract [35]. These results may indicate that FPP^®^ could act as a macrophage activator because of its augmentation on nitric oxide synthesis and on TNF-α secretion.

It is known that the deposition of the substance β-amyloid (Aβ) is related to Alzheimer’s disease development although its exact action mechanisms are not yet well defined. Studies show that there may be an association between free radicals and the development of Alzheimer’s disease. Hence, due to its antioxidant properties, the effect of FPP^®^ on reducing the Aβ associated with Alzheimer’s disease was evaluated by Zhang and collaborators (2006). In their analysis, a model of cellular Alzheimer’s disease was used by using copper to trigger the neurotoxicity of Aβ. About the accumulation of reactive oxygen species (ROS), copper increased the ROS generation that was eliminated when cells were post-treated with FPP^®^, protecting the cell from the Aβ neurotoxicity. This treatment also decreased the lipid peroxide level and the accumulation of intracellular nitric oxide (NO). These authors found that FPP^®^ can improve antioxidant effect in vivo and in vitro by increasing free radical scavenging. FPP^®^ has also decreased the accumulation of ROS and NO. In fact, FPP^®^ can improve Aβ neurotoxicity impaired by copper and prevent cell apoptosis [36].

Marotta and co-workers (2006) conducted a placebo-controlled and randomized cross-over study to examine the FPP^®^ effects on redox activity and DNA damage in a healthy elderly population. The elderly population has less antioxidant capacity due, for example, to nutritional deficits, reduced nutritional intake, and failures in intestinal absorption. Dietary antioxidants can reduce DNA adducts depending on the detoxifying activity of GSTM1 isoenzyme. Therefore, these authors analyzed the polymorphism of GSTM1 (glutathione S-transferase M1) in 54 elderly individuals. One group was orally supplemented with FPP^®^ (9 g/day), and the control group received the same amount of placebo (flavored powdered sugar) for three months, followed by a six-week washout period. The FPP^®^ oral supplemented group showed a significant enhancement of the antioxidant protection in the GSTM1 negative subgroup regardless of smoking (*p* < 0.01). It was also observed that FPP^®^ exerts protective effects on leukocyte DNA adducts formation irrespective of genotype profile. Additionally, FPP^®^ oral supplementation can enhance DNA repair mechanisms but only in GSTM1-null genotype patients. These results showed that FPP^®^ can improve antioxidant activity in elderly individuals even without any overt antioxidant deficiency state [37]. In order to continue the previous study’s conclusions, Marotta and his colleagues (2010) studied the effect of FPP^®^ on redox balance gene expression in 11 healthy non-smoker subjects. All patients were sublingually supplemented with FPP^®^ 6 g/day, and the blood was collected at the second and fourth weeks of observation. The researchers analyzed the antioxidant enzyme activity, lipid peroxidation, and protein oxidation. They observed that the plasma level of the tested redox parameter was unchanged, but FPP^®^ significantly upregulated all tested gene expression (*p* < 0.05) [38]. These results, although preliminary, indicate the nutrigenomic potential of FPP^®^.

The effect of FPP^®^ on the type IV allergic reactions in skin and intestine was investigated by Hiramoto and his colleagues (2008). Th1-dependent type IV and Th2-dependent mucosal allergic reactions were elicited in the skin and intestine of male ICR mice by repeated sensitization with oxazolone and fluorescein isothiocyanate (FITC), respectively.

In this study, it was observed that plasma levels of IFN-γ, IL-10, and TNF-α, measured by ELISA, increase either FITC or oxazolone sensitization and that, after FPP^®^ administration, they were reduced. Both FITC and oxazolone increased ear and colon thickness at 24 h after the challenge. After either the FITC or oxazolone challenges, the colonic expression of IgA markedly increased, but there was a little expression in the FPP^®^-treated animals in comparison to the dextrose-treated animals. Biochemical analyses revealed that the FPP^®^ decreased ear swelling, inflammatory cytokine levels in plasma, and IgA, dendritic cells, and inflammatory cells expression in colon [39]. These results suggest that FPP^®^ may have therapeutic potential for the prevention of contact hypersensitivity.

Mammary gland hyperplasia is a common noninflammatory and nontumor disease that occurs in women of child-bearing age. This condition accounts for 75% of all breast diseases. The occurrence of mammary gland hyperplasia is directly related to endocrine disorders, which are mainly caused by an imbalance of estrogen and progestin. Accordingly, a study investigated the protective effect, functions, and action mechanism of Fermented Papaya Extracts (FPEs) obtained through *Aspergillus* and yeast long-term fermentation in estrogen-induced mammary gland hyperplasia. The study was performed using 36 female rats divided into six groups: the blank control group, the FPEs control group, the model group (that received an intramuscular injection of estradiol benzoate and of progestin), and the three treatment groups (that received estradiol benzoate and progestin equal to the model group and were treated orally with FPEs in three different concentrations). After 30 days, the right nipple of the rats in each group was shaved for the measurement of the diameter and height. Blood and tissue samples were collected from the mammary glands, heart, liver, spleen, lung, kidney, ovary, and uterus, and the concentration of each sex hormone (estradiol, progesterone, luteinizing hormone, and follicle-stimulating hormone) was analyzed. Histopathological analysis showed that the blank control group and the control group administered with only FPEs showed no apparent hyperplasia. Instead, severe pathological changes (mammary gland hyperplasia, lobule increase, acinar increase, mammary duct and lumen ectasia, and mammary duct and lumen secretion increase) were observed in the model group. Analysis of oxidant levels showed that FPEs may increase superoxide dismutase (SOD) and glutathione peroxidase (GSH-Px) activities and decrease malondialdehyde (MDA) [40]. The results indicated that FPEs can increase the antioxidant levels and inhibit the hyperplasia of mammary glands by antioxidants and prevent liver damage via their antioxidant properties.

Fujita and his collaborators (2017) examined the effects of FPEs supplementation for 30 days on immunological and metabolic functions and fecal flora in tube-fed patients. Study participants were divided into three groups: a control group (that did not receive FPE), and two treated groups (administered with 3 g FPE per day or 9 g FPE per day). Elderly patients who are long-term tube-fed have decreased peripheral blood mononuclear cell (PBMC) cytolytic activity. Administration of FPE at 3 and 9 g per day restored PBMC cytolytic activity, significantly increased NK cell cytotoxicity in a dose-dependent manner, and did not affect IgG, IgA, and IgM levels. Analysis of fecal samples at the beginning of the study, in the control group, and also in the FPE groups showed characteristic microbiota with a high proportion of phylum *Firmicutes* and genus *Parabacteroides* and with a low proportion of genus *Bifidobacterium*. Administration of FPE at 9 g per day reduced the abundance of *Firmicutes*, *Clostridium scindens*, and *Eggerthella lenta*, and the fecal odor [41].

Yoshino and co-workers (2009) investigated the ability of long supplementation (5–7 months) of FPP^®^ to modulate oxidative stress following the electron spin resonance images of the 3-methoxycarbonyl-2,2,5,5-tetramethylpyrrolidine-1-oxyl (MC-PROXYL). The supplementation with FPP^®^ led to a rapid decay of MC-PROXYL in the spontaneously hypertensive rat (SHR) brain. The decay rate constant of MC-PROXYL in the isolated brains of SHR is increased in the brain of normal male Wistar Kyoto rats. Supplementation with FPP^®^ increased the decay rate constant of MC-PROXYL in the isolated brains of SHR. This appears to indicate that FPP^®^ up-regulated antioxidant activity in the SHR brain. It can be concluded that FPP^®^ has protective effects against oxidative injury, supporting the view that prophylactic potentials in chronic degenerative diseases and particular diseases of overt inflammation [35].

Another study from Murakami and collaborators (2016) analyzed FPP^®^ effects in carcinogenesis in vivo and immunomodulatory function in vitro. They reported an anti-tumor effect of FFP^®^ in vivo using mouse cancer models. Oral administration of FPP^®^ inhibited tumor growth, and a dose of 450 mg/kg/day eliminates the tumor. Interleukin-1β (IL-1β), TNFα, and IFNγ, released by immunocytes, participate in numerous immunological and inflammatory reactions and can inhibit tumor progress. Thus, this work stated that FPP^®^ can activate innate immunity and has chemotherapeutic properties, by stimulating IL-1β, TNFα, and IFNγ released in vitro. The authors also observed that oral administration of FPP^®^ inhibited tumor evolution in mouse cancer models in a dose-dependent manner. Additionally, oral administration of FPP^®^ a dose of 450 mg/kg/day results in complete disappearance of the tumor. However, these results were only observed after oral but not intraperitoneal administration [42]. This may happen due to FPP^®^ metabolism and interactions with microflora.

A recent in vivo study by Logozzi and co-workers (2019) reported that FPP^®^ can control murine melanoma tumor growth and reduce a tumor mass of about 3–7 times versus untreated mice. They verified that the optimal effect of FPP^®^ on tumor growth was with a dose of 200 mg/kg/day. They also demonstrated a decrease in the blood total ROS levels and an increase in levels of antioxidants free glutathione (GSH) and enzyme superoxide dismutase-1 (SOD-1). These results are promising in tumor prevention and antioxidant effects [43]. These results show great perspectives and can lead to new studies about FPP^®^ as an anticancer agent.

Another recent study from Logozzi et al. (2020) suggested the FPP^®^ has anti-aging and antioxidant effects. Mice were daily treated with FPP^®^ and compared with mice receiving only tap water. After ten months of the FPP^®^ treatment, it was shown that FPP^®^ induced an increase in telomeres length in bone marrow and ovary. Beyond this, they also caused an increase in the plasmatic levels of telomerase activity and the antioxidant levels. This study has also evaluated the FPP^®^ effect on redox balance, with a decrease of ROS, and with an anti-aging effect, as shown by the length of telomeres and telomerase quantification in FPP^®^-treated mice. This study also showed that FPP^®^ was more effective when it starts at an early age as compared to late treatment. The results suggest also that FPP^®^ treatment may extend the fertility period at least in the females, suggesting that the use of FPP^®^ may be helpful in preventing or treating female infertility [44].

In general, fermented papaya has the ability to modulate the immune system and exhibits antioxidant activity, being able to reduce ROS, but also improves redox balance and boosts natural defense mechanisms of the immune system. Fermented papaya has also shown the capacity to inhibit tumor growth. Fermented papaya’s antioxidant activity may be attributed to its polyphenolic content. However, as a natural product, the active compounds responsible for the described benefits may vary due to several reasons, such as the fruit cultivation conditions (country, temperature, and soil quality) and the fermentation protocol (temperature, duration, and used strains). In addition, it is reported that fermented papaya has low molecular weight oligosaccharides different from fresh fruit, which may be responsible for the immunomodulatory activity of fermented papaya [45].

### 3.2. Congenital/Acquired Haemolytic Anemias

The results of the studies regarding fermented papaya health benefits in congenital/acquired hemolytic anemias are summarized in Table 2.

Thalassemias are a group of inherited diseases that lead to malfunctioning hemoglobin production. Patients with thalassemia have a mutation that causes inefficient erythropoiesis [46]. Iron-induced toxicity in β-thalassemia is the most important cause of oxidative stress [47]. Data from some studies suggest that FPP^®^ has antioxidant properties that may have some benefit in hemoglobinopathies, such as β thalassemia. In a study that was set out to determine the FPP^®^ effect in β-thalassemia, Amer and his colleagues analyzed the antioxidant effects of FPP^®^ in vitro and in vivo on red blood cells (RBC), platelets, and polymorphonuclear of β-thalassemia mice and patients. For this, mice were treated orally with FPP^®^ (50 mg/mouse/day for three months) and 11 patients (eight β-thalassemia intermedia and three β-thalassemia major) with 3 g of FPP^®^ three times a day during the same period. The current study started to find that in vitro FPP^®^ treatment reduced thalassemic red blood cell sensitivity to hemolysis and phagocytosis, improved polymorphonuclear ability to generate oxidative burst, and reduced platelet tendency to undergo activation. Furthermore, the researchers studied FPP^®^ in vivo effect in thalassemic mice and observed that treatment with FPP^®^ significantly reduced all tested oxidative stress parameters. Finally, they studied the FPP^®^ effect in patients, and like the results obtained in vitro, these results showed a significant reduction in all oxidative stress parameters tested in blood cells [48]. Another study conducted by some authors of the previous study investigated the effect of oral supplementation with FPP^®^ in oxidative status of two groups of thalassemia patients: β-thalassemia major and intermedia (in Israel) and E-β-thalassemia (in Singapore). The researchers reported that oral supplementation with FPP^®^ showed a significant decrease in all oxidative stress parameters tested in their RBC [49]. The evidence presented before suggests that FPP^®^ has a potent antioxidant power and might relieve symptoms associated with oxidative stress in severe forms of thalassemia. The antioxidant effect of FPP^®^ in reducing oxidative stress biomarkers in these studies is undetermined although one possible justification could be its high content of glutamic acid, glycine, and methionine, which are substrates for reduced glutathione (GSH) production [44]. Despite this, both previous studies showed no significant improvement in the hematological parameters [43,44].

A report analyzed by Ghoti and his colleagues presents a clinical case regarding a patient with paroxysmal nocturnal hemoglobinuria, an acquired blood disease characterized by intravascular hemolysis, which is the main cause of anemia. After oral supplementation with FPP^®^ (3 g, 3 times/day, for 4 months), this patient’s free hemoglobin (Hb), white blood cell, and all other hemolytic parameters levels increased. They also described a significant decrease of the malondialdehyde level (a product of lipid membrane peroxidation). Overall, there seems to be some evidence that FPP^®^ treatment has an antioxidant effect and seems to benefit in paroxysmal nocturnal hemoglobinuria [50]. Following up on the discoveries of the previous report and to better understand the FPP^®^ antioxidant effect in hemoglobinopathies, Ghoti and his colleagues developed an in vivo and in vitro study to evaluate the FPP^®^ effect in hereditary spherocytosis (HS). The in vitro study was performed with RBC from seven HS patients. They treated RBC-HS with FPP^®^ (0.1 mg/mL for 2 h) and observed significantly reduced oxidate stress markers, such as a decrease in ROS, an increase in reduced glutathione, and less hemolysis in RBC treated with FPP^®^. The in vivo study included ten patients with mild to severe HS and supplemented them with 3 g of FPP^®^ 3 times/day for 3 months. After 3 months, FPP^®^ increased hemoglobin levels from 11.2 to 12.4 g/dL and mean corpuscular hemoglobin concentration decreased from 34.5 to 33.4 g/dL. They also verified a significant decrease in lactate dehydrogenase from 550 to 458 U/L [51]. Thus, these series of promising results suggest FPP^®^ has a potent antioxidant effect, which can relieve symptoms associated with oxidative stress in both congenital and hemolytic anemias [44,45,46].

In line with the previous topic, FPP^®^ reduces oxidative stress, decreases ROS, and improves immune system defenses. FPP^®^ can protect red blood cells, which therefore improves hemolytic parameters and may be useful in the treatment/control of symptoms of congenital or hemolytic anemia. This health benefit can be justified based on the nutritional richness of papaya in vitamins or minerals.

### 3.3. Antidiabetic and Antidislipidemic Properties

Diabetes mellitus is a metabolic disease described by hyperglycemia and insufficiency of insulin. Oxidative stress is considerable as an important contributing factor in the pathogenesis of type 2 diabetes by an excess of ROS and glucose autoxidation [52]. Danese and his colleagues (2006) showed that oral supplementation with FPP^®^ causes a significant decrease in plasma glucose levels in both healthy and type 2 diabetes patients. As such, they divided 50 individuals into two groups: a group of 25 patients diagnosed with type 2 diabetes mellitus undergoing pharmacological treatment with an oral antidiabetic and a control group of 25 healthy people. All individuals were supplemented with oral FPP^®^ (3 g/day) for three months. They observed a hypoglycemic effect in both groups, concluding that FPP^®^ can be used as an adjunct therapy in type 2 diabetes mellitus [53].

Additionally, FPP^®^ supplementation improved the blood lipid profile: total triglycerides, total cholesterol, and low-density lipoproteins levels decreased significantly (*p* < 0.05), while high-density lipoproteins levels (HDL) increased [54]. In fact, intake of individual antioxidants has been related to a lower risk of type 2 diabetes [55]. In addition, intake of carotenoids, such as β-carotene, has been reported due to the ability to reduce type 2 diabetes risk [56]. Carotenoids, which are widely present in papaya, might play a role in the treatment of diabetes mellitus and its side effects. Most reliable studies confirm that there is an inverse correlation between the plasma carotenoid concentration and diabetes mellitus incidence [57]. Furthermore, as oxidative stress is present in diabetes (due, for example, to glucose autoxidation), the antioxidant capacity of fermented papaya appears to have a protective action. Oxidative stress is increased in diabetes by the glucose auto oxidation and low levels of antioxidants. Thus, oxidative stress contributes to diabetes or its resulting complications. As fermented papaya exhibits antioxidant properties, it permits to reduce oxidative stress and therefore controls diabetes and even other dyslipidemias.

### 3.4. Skin Benefits and Wound-Healing Properties

The results of the studies regarding the fermented papaya health benefits in skin and wound-healing properties are summarized in Table 3.

Mikhal and co-workers (2004) conducted a study to evaluate in vivo and in vitro wound-healing effect of the preparation from fermented papaya sold as BioRex^®^. They induced burn trauma in Wistar rats (burns IIA-B-20% of skin area) and evaluated the effects of the preparation from fermented papaya. The control group was treated with paraffin gauze dressings. The antioxidant effect of BioRex^®^ in skin was tested in vitro, analyzing the formation of the hydroxyl and superoxide radicals (H_2_O_2_—FeSO_4_) and xanthine (xanthine oxidase model), and there was an observed antioxidant effect on treated cells (human peripheral blood neutrophils). In vivo studies (thermal wound model in rats), with BioRex^®^ topical treatment reduced the wound area and bacterial burden (*Staphylococcus aureus*) by lowering their catalase level. This study also revealed considerable differences in the rate of wound healing with BioRex^®^ when comparing treated and control rats on day 8 of the experiment. On day 12 after trauma, the wound area in BioRex^®^-treated rats was two-fold lower than in control animals. The results suggest that local treatment with the preparation from papaya accelerates wound healing in rats with burn trauma. This preparation decreased the production of free radicals in the whole blood of animals, which reflects changes in the general inflammatory reaction related to the indirect antibacterial effect and antioxidant properties [58]. The beneficial actions of papaya on the skin may be also due to the presence of enzyme papain that may facilitate wound debridement [60].

Collard and Roy (2010) studied the effects on wound healing in adult obese diabetic mice. FPP^®^ supplementation significantly improved wound healing in diabetic mice. They also verified an improved nitric oxide production by wound macrophages and elevated iNOS gene expression in wound tissue in FPP^®^-supplemented diabetic mice. It is known that in diabetes, there is a deficit of NO in the wound site, and it is also known that NO is involved in wound healing through multiple modes of action. Hence, an increase in NO delivery by FPP^®^ has a beneficial effect on diabetic wound healing [54]. These results confirm the previously reported study from Danese and his colleagues (2006). In fact, recent research has shown that oral supplementation with FPP^®^ causes a decrease in plasma sugar and improves the blood lipid profile [53]. It is also known that oxidative stress is increased in diabetes by glucose autoxidation and low levels of antioxidants [61].

A double-blind study aimed to compare the skin anti-aging effect of oral supplementation between FPP^®^ and an antioxidant cocktail (10 mg trans-resveratrol, 60 µg selenium, 10 mg vitamin E, and 50 mg vitamin C). For this study, 60 healthy non-smoking individuals between 40 and 65 years old with clinical signs of skin aging were recruited. In the FPP^®^-treated group, patients were sublingually supplemented with 9 g/day (4.5 g × 2 times/day). In the antioxidant control group, patients received an antioxidant cocktail in the same way as in the other group. In both groups, the FPP^®^ and the antioxidant cocktail administration was performed for 90 days. The authors measured parameters, such as skin moisturization, skin elasticity, skin surface, brown spot intensity, skin gene expression, dermal redox balance, and nitric oxide assessment. They observed that FPP^®^ can increase skin moisturization after 90 days (~95% increase; *p* < 0.04), and the antioxidant cocktail did not change this parameter. The overall evenness was assessed as significantly enhanced in the FPP^®^-treated group (*p* < 0.05) but not in the antioxidant-control group. Both FPP^®^ and antioxidant cocktail improved the malondialdehyde and superoxide dismutase skin levels, but only the FPP^®^-treated group exhibited a higher superoxide dismutase level and a significant nitric oxide rise. They also observed a significant upregulation of defensive genes and downregulation of the potentially pro-aging/carcinogenic genes [59].

In accordance with previous sections, the high antioxidant power of fermented papaya, its anti-inflammatory capacity, and the ability to reduce free radicals have proved to be useful on skin, specifically in wound healing. Chronic wounds in patients with diabetes represent a major public health problem. Macrophages at the wound site of patients with diabetes are compromised in their ability to support wound healing. Fermented papaya may improve diabetic wound outcomes by specifically influencing the macrophage response at the wound site and subsequent angiogenic response. In addition, fermented papaya is also useful in hydrating the skin. This fact, combined with its antioxidant properties, shows that fermented papaya can be an excellent natural anti-aging agent.

## 4. Conclusions

Fermented papaya is recognized as a nutraceutical with an exceptionally diverse composition. Although only the pulp of the fruit is consumed, both the peel and the seeds of the papaya have a great phytochemical value, so they should not be wasted. Several experiments to determine its biological activity were carried out. Several studies have proven its anti-inflammatory, immunomodulatory, anticancer, and antioxidant physical activities. Intake of antioxidants and nutraceuticals reduces oxidative stress and can help mitigate and prevent various illnesses, such as Alzheimer’s disease and dementia. Fermented papaya has several health benefits, highlighting its antioxidant potential. Cellular damage induced by oxidative stress has been implicated in a variety of chronic diseases, such as cancer and neurodegenerative disorders. The antioxidant action of papaya is also very relevant in dermo-cosmetics, as many skincare creams have anti-aging action. The beauty and health of the skin, hair, and nails are directly linked to a diet rich in vitamins and minerals, so this fruit has many benefits at this level. The anti-inflammatory power of papaya is useful in reducing inflammation and oxidation parameters. Its anti-tumor action is also noteworthy.

Given the main nutritional and health applications of fermented papaya described in the literature, this review aimed to emphasize the importance of early daily intake of a very potent antioxidant compound, such as fermented papaya, to possibly eliminate free radicals and control the aging process and prevent the development of various diseases. However, an in-depth investigation based on its pharmacokinetic properties as well as clinical trials is required. Further studies are also needed to know the exact composition of fermented papaya supplements and which compounds are responsible for their actions. In addition, the availability of a huge variety of fermented papaya supplements requires a review of the legal provisions for drug manufacturing. Hence, it is important that the agencies responsible for the supplement’s commercialization verify the compliance of fermented papaya supplements. There is already a brand with many in vivo and in vitro studies (FPP^®^). However, not all brands available on the market can be produced with the same fermentation procedure.

## Figures and Tables

**Table 1:** Immunomodulatory, antioxidant, and anticancer properties of fermented papaya.

Preparation	Study Type	Dose	Model	Bioactive Effect	Reference
FPP^®^	In vitro study	3 mg/mL	Murine monocyte/macrophage cell line RAW 264.7 treated with IFN-γ and/or FPP^®^	FPP fractions in the presence of IFN-γ both LMF and HMF: ↑ iNOS activity ↑ nitrate and nitrite accumulation	[35]
FPP^®^	In vitro study	1.2, 2.4, 4.8 mg/mL	AD cell model	Attenuated the Aβ neurotoxicity and prevents the cell apoptosis ↓ ROS ↓ accumulation of intracellular NO ↓ iNOS	[36]
FPP^®^	Randomized, Placebo-Controlled, Cross-Over Study	Group A: 9 g/day Group B: placebo (3 months)	54 elderly patients randomly divided into 2 groups (Group A and B)	FPP^®^ supplemented group: ↑ antioxidant protection	[37]
FPP^®^	Pilot study	6 g/day	11 healthy nonsmoker patients	Plasma level of the tested redox parameter did not change ↓ nonsignificant decrease of MDA upregulation of all gene expression investigated	[38]
FPP^®^	In vivo study	6 mg/mouse/day	Specific pathogen-free male ICR mice with 8 weeks old	↓ contact hypersensitivity ↓ IFN-γ, IL-10, and TNF-α ↓ IgA and dendritic cells	[39]
FPEs	In vivo study	5–30 mL FPEs/kg (1 month)	36 female mice divided into 6 groups	Protective effect of FPEs on mammary gland pathology in model animals: ↑ SOD and GSH-Px ↓ MDA	[40]
FPEs	Clinical study	Group A: control group Group B: 3 g/day Group C: 9 g/day (1 month)	4 males and 8 females with cerebrovascular disease, 4 males and 3 females with neurodegenerative disease, and 1 male post-traumatic head injury divided into 3 groups	↑ PBMC cytolytic activity ↑ NK cell cytotoxicity ↓ *Firmicutes*, *C. scindens,* and *E. lenta* ↓ offensive fecal odor	[41]
FPP^®^	In vivo study	50 mg/mouse/day (5–7 months)	SHR	↓ MC- PROXYL ↑ redox defense activity in SHR brain	[35]
FPP^®^	In vivo study	150, 300, or 450 mg/ kg/day (oral administration)/ 1600 or 4000 mg/kg/2 days (intraperitoneal injection)	Mouse cancer model	Oral administration of FPP inhibited tumor growth	[42]
	In vitro study	10 mg/mL	PBMC	↑ IL-1β, TNFα and IFNγ	
FPP^®^	In vivo study	200, 400 mg/kg/day	35 mice cancer models divided into 7 groups	↓ tumor mass of about three to seven times vs. untreated mouse; ↓ ROS ↑ antioxidants (SOD-1 and GSH)	[43]
FPP^®^	In vivo study	6 mg/mouse/day	40 aging female mice model divided into 2 groups	↑ telomerase activity ↓ ROS ↑ antioxidants	[44]

Aβ, substance β-amyloid; AD, Alzheimer’s disease; FPEs, fermented papaya extracts; GSH, free glutathione; GSH-Px, glutathione peroxidase; HMF, high molecular fraction (FPP^®^); IFN-γ, interferon- γ; IL-1β, interleukin-1β; iNOS, nitric oxide synthase; LMF, low molecular fraction (FPP^®^); MG, methylguanidine; NO, nitric oxide; PBMC, human peripheral blood mononuclear cells; ROS, reactive oxygen species; SHR, spontaneously hypertensive rats; SOD, superoxide dismutase; SOD-1, enzyme superoxide dismutase-1; TNF-α, tumor necrosis factor-α. ↑ increase, ↓ decrease.

**Table 2:** Fermented papaya health benefits in congenital/acquired hemolytic anemias.

Preparation	Study Type	Dose	Model	Bioactive Effect	Reference
FPP^®^	In vitro study	10 mg/mL	Normal and thalassemic RBC	↑ glutathione content of RBC, platelets, and PMN leukocytes ↓ ROS and membrane lipid peroxidation	[42]
	In vivo study	50 mg/mouse/day (3 months)	β-thalassemia mice models		
	Observational study	3 g 3 times/ day (3 months)	8 patients with β -thalassemia intermedia and 3 with β -thalassemia major		
FPP^®^	Observational study	3 g 3 times/day	β- thalassemia group (8 patients with β-thal intermedia and 3 with β-thal major)	↑ GSH ↓ ROS; membrane lipid peroxidation	[43]
		3 g 2 times/day	E-β- thalassemia group (7 patients)		
FPP^®^	Case report	3 g 3 times/day (4 months)		↑ Hb level ↓ LDH and Malondialdehyde ↓ patient fatigue ↑ patient performance	[44]
FPP^®^	In vitro study	Incubation of HS-RBC for 2 h with 0.1 mg/mL	HS-RBC from 17 patients	↑ Hb level ↓ Malondialdehyde ↓ ROS ↑ GSH	[45]
	In vivo study	3 g 3 times/day (3 months)	10 (8 males and 2 females) HS patients		

FPPP^®^, fermented papaya preparation; GSH, reduced glutathione; Hb, free hemoglobin; HS, hereditary spherocytosis; LDH, lactate dehydrogenase; PMN, polymorphonuclear; RBC, red blood cells; ROS, reactive oxygen species. ↑ increase, ↓ decrease.

**Table 3:** Fermented papaya skin benefits.

Preparation	Study Type	Dose	Model	Bioactive Effect	Reference
BioRex^®^	In vitro study	1–5 mg/mL	Peripheral blood neutrophils	Suppressed generation of superoxide, hydroxyl radical, and total production of radicals; ↓ catalase activity	[58]
	In vivo study	-	Thermal wound model in rats	↓ wound area and bacterial burden; ↓ catalase activity	
FPP^®^	In vivo study	FPP group: 0.2 g/kg 5 days/week (8 weeks) Placebo group: D-glucose in the same manner as in FPP group (8 weeks)	Adult obese diabetic mice divided into 2 groups (FPP and placebo control)	↑ NO and iNOS	[54]
FPP^®^	Double-blind study	FPP group: 4.5 g 2 times/day (90 days) Antioxidant control group: Antioxidant cocktail with similar flavored and in the same manner as in FPP group (90 days)	60 healthy non-smoker males and females (40–65 years) divided into 2 groups	FPP supplementation: ↑ skin moisturization, evenness, and elasticity; ↑ NO and SOD production; Gene regulatory improvement in the skin.	[59]

FPP^®^, fermented papaya preparation; NO, nitric oxide; SOD, superoxide dismutase. ↑ increase, ↓ decrease.

## Data Availability

Not applicable.
